# Differences in Static and Dynamic Resting-State Functional Connectivity between Migraineurs with and without Photophobia, without Phonophobia or Osmophobia

**DOI:** 10.3390/neurosci5030017

**Published:** 2024-06-23

**Authors:** Noboru Imai, Asami Moriya, Eiji Kitamura

**Affiliations:** 1 Department of Neurology, Japanese Red Cross Shizuoka Hospital, Shizuoka 420-0853, Japan; 2 Department of Neurology, Kitasato University, Sagamihara 252-0373, Japan

**Keywords:** migraine, photophobia, functional connectivity, cerebellar hemisphere, temporal region

## Abstract

Background: We have previously shown that static and dynamic resting-state functional connectivity differ between migraineurs with and without photophobia, phonophobia, or osmophobia. Furthermore, some patients with photophobia also experience phonophobia or osmophobia. To investigate the functional connectivity specific to migraineurs with photophobia, we examined the differences in static and dynamic resting-state functional connectivity between patients with and without photophobia, with no phonophobia or osmophobia. Methods: Fifteen migraineurs with photophobia but without phonophobia or osmophobia, as well as 15 sex- and age-matched migraineurs without photophobia, phonophobia, or osmophobia, underwent 3-T functional magnetic resonance imaging during the interictal phase. Static and dynamic resting-state functional connectivity were compared using region-of-interest analyses of 91 cortical, 15 subcortical, and 26 cerebellar areas. Results: Static resting-state functional connectivity analysis revealed ten significant connectivity pairs in patients with photophobia, while dynamic resting-state functional connectivity analysis revealed six significant connectivity pairs in patients with photophobia. Migraineurs with photophobia had significantly lower connectivity between the cerebellar hemisphere and the temporal region than those without photophobia in both static and dynamic studies. Conclusions: Our results show that lower resting-state functional connectivity between the cerebellar hemisphere and the temporal region is specific to migraineurs with photophobia.

## 1. Introduction

Photophobia is one of the most common symptoms of, and is a recognized diagnostic criterion for, migraine [1]. A recent study showed that photophobia was the most bothersome symptom (MBS) in many patients, and that patients reporting photophobia as the MBS were more likely to have cutaneous allodynia, and less likely to have a visual aura [2]. However, the differences in pathogenesis between patients with and without photophobia have not yet been fully investigated.

Functional magnetic resonance imaging (fMRI) has previously been used to investigate the mechanisms leading to sensory hypersensitivity in migraines by measuring brain responses to sensory stimulation. Functional connectivity analyses have further investigated the functional organization of specific brain regions and networks responsible for sensory processing [3,4]. Functional connectivity describes the temporal correlation between spatially separated neurophysiological events, often measured using techniques such as functional magnetic resonance imaging (fMRI), electroencephalography (EEG), or magnetoencephalography (MEG) [3,4]. Functional connectivity using MRI is often assessed by examining the synchronization of blood oxygen level-dependent signals in different brain regions [3,4]. There are two types of functional connectivity: static and dynamic [5]. Static connectivity assesses average connectivity patterns over a fixed period. Functional connectivity was assumed to be stable during the observation period. Conversely, dynamic connectivity examines how connectivity patterns change over time, recognizing that functional connectivity may fluctuate with cognitive state or task.

We have previously shown that static and dynamic resting-state functional connectivity differ between migraineurs with and without photophobia, phonophobia, or osmophobia. In addition, patients with photophobia show significantly different connectivities, particularly between the cerebellar lobules and other brain regions [5]. In a previous study, we encountered limitations in assessing the classification accuracy of different subgroups of migraineurs, particularly because of the co-occurrence of photophobia with phonophobia or osmophobia in some patients. To delve deeper into the functional connectivity unique to migraineurs with photophobia, our study focused on detecting disparities in both static and dynamic resting-state functional connectivity between patients with and without photophobia, while excluding individuals with concurrent phonophobia or osmophobia.

## 2. Materials and Methods

### 2.1. Study Design and Participants

Study participants were enrolled from the Department of Neurology at Japanese Red Cross Shizuoka Hospital. The participants were aged between 18 and 65 years, and all patients fulfilled the International Classification of Headache Disorders, 3rd edition, criteria for migraine, with or without aura, as determined by a headache specialist certified by the International Headache Society. The exclusion criteria were as follows: tension-type headache lasting for more than five days per month, history of any other primary headache, pregnancy, breastfeeding, contraindication for MRI, any cardiovascular or cerebrovascular disease, uncontrolled psychiatric disorder, or drug abuse. The study protocol was approved by the Institutional Review Board (Approval number 2017-08), and was conducted in accordance with the Helsinki II Declaration of 1964 and its subsequent revisions. Written informed consent was obtained from all participants. Physical and demographic data, including body mass index (BMI), age, and sex, were collected from all participants.

### 2.2. Statistical Analyses of Demographic and Clinical Variables of Patients

Variables common to the patients were analyzed using *t*-tests for continuous data and Fisher’s exact test for classified data using SPSS Statistics (version 20.0; IBM Corp., Armonk, NY, USA). The significance level was set at *p* < 0.05.

### 2.3. MRI Acquisition

#### 2.3.1. Functional Images

All images were acquired using a 3.0-T General Electronic Healthcare Discovery scanner (Chicago, IL, USA) during the interictal phase. A resting-state echo-planar imaging scan (40 axial slices; 3.75 × 3.75 mm in-plane resolution; slice thickness, 4.0 mm; 250 volumes; and repetition time/echo time (TR/TE), 2500/40 ms) was acquired for each patient. The parameters were as follows: TE, 40 ms; TR = 25,000 ms; field of view (FOV), 250 mm; flip angle, 90°; matrix size, 64 × 64; number of axial slices = 40; and voxel size = 3.75 × 3.75 × 4.00 mm^3^. Patients were instructed to rest with their eyes closed without falling asleep during the scan. 

#### 2.3.2. Structural Images

High-resolution T1-weighted fast-field echo structural scanning was performed. The details of the parameters were as follows: TE = 3.7 ms, TR = 8.0 ms, flip angle = 15°, matrix size = 160 × 256 × 256, and voxel size = 1 × 1 × 1 mm^3^.

#### 2.3.3. Preprocessing, Static and Dynamic Functional Connectivity Analysis

RS-fMRI preprocessing was completed using MATLAB R2015b (MathWorks Inc., Sherborn, MA, USA), Statistical Parametric Mapping software (SPM12, Wellcome Trust Centre for Neuroimaging, University College London, UK), and functional connectivity toolbox version 17 (CONN) [6]. We further compared static and dynamic resting-state functional connectivity using ROI-to-ROI analysis of 91 cortical, 15 subcortical, and 26 cerebellar areas identified a priori using the CONN toolbox. The experimental protocol for preprocessing and static and dynamic functional connectivity analyses was conducted in accordance with the methods described in our previous study [5]. In brief, static functional connectivity analysis was conducted through ROI-to-ROI analysis using the CONN toolbox [5,6]. In this approach, the target voxel BOLD time series S(x, t) was replaced by the target ROI time series Rj(t). The resulting ROI-to-ROI correlation matrices illustrate the functional connectivity levels between each ROI pair. The ROI-to-ROI correlation was determined using the Fisher-transformed bivariate correlation coefficient between the BOLD time series of the two ROIs. Dynamic independent component analyses were performed for the dynamic FC using the CONN toolbox [5,6]. These analyses investigated the temporal modulation properties of the ROI-to-ROI connectivity matrix to uncover circuits with similar modulated connections. Dynamic independent component analysis (ICA) matrices quantified the expression of different modulatory circuits and the connectivity change rate between ROI pairs, as indicated by the connectivity strength and sign variations co-varying with a specific component/circuit time series. Group-level modulatory components Gamma_l(i, j) were computed through several steps. First, a simplified generalized context-dependent psychophysiological interaction (gPPI) model was used to estimate the group-level modulatory components Gamma_l(i, j). These components were rotated using fastICA with a hyperbolic tangent contrast function. The ICA mixing matrix W was inverted to derive the dynamic IC/circuit time series. Finally, the group-level modulatory components were back-projected onto the subject-level components gamma_nk(i,j) via a series of standard first-level gPPI models, incorporating the estimated dynamic independent component/circuit time series h(t) as gPPI psychological factors. The results of effective connectivity are reported when significant at a level of *p* < 0.05, with the corrected false discovery rate (FDR).

## 3. Results

### 3.1. Participants

Data from 30 patients with migraine with or without aura were analyzed in this study. Fifteen patients with migraine had photophobia, but not phonophobia or osmophobia. The remaining 15 sex- and age-matched patients with migraine did not exhibit photophobia, phonophobia, or osmophobia. In both subgroups, as well as in the total group, 73% of the patients were female. The average age was approximately 40 years across all groups, with minor variations, with an overall average age of 40.4 years. Those with and without photophobia had average ages of 40.5 and 40.3 years, respectively (*p* = 0.67). This study analyzed the demographic characteristics of participants, revealing an average migraine history duration of 22.9 years overall, 22.3 years with photosensitivity, and 23.4 years without photosensitivity, with no significant differences observed between the two groups (*p* = 0.529). Additionally, the average BMI was 22.1, 22.0, and 22.3, respectively, with no significant differences detected between the groups (*p* = 0.724).

None of the patients had a history of medication overuse headache.

### 3.2. Differences in Static Resting-State Functional Connectivity

**Figure 1:**
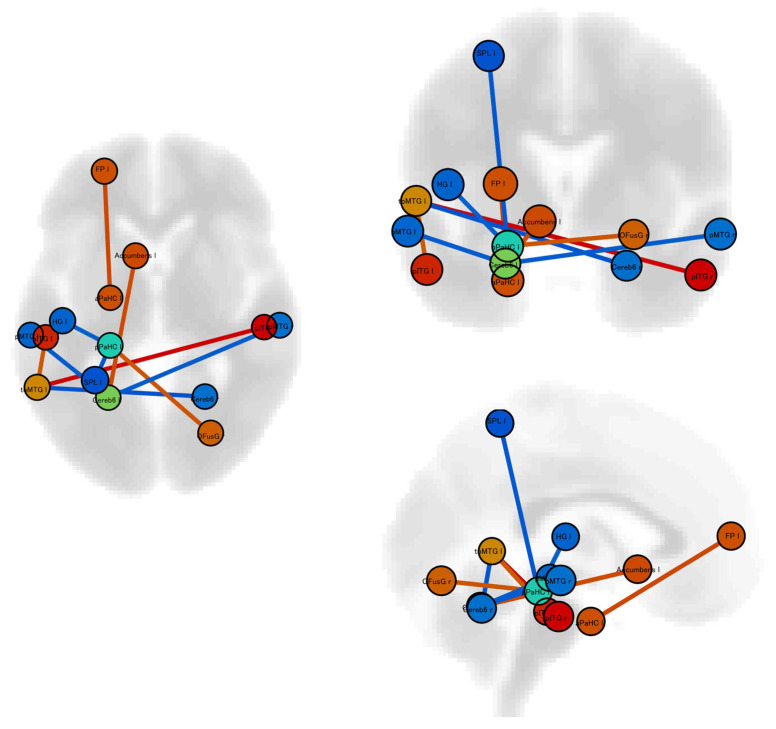
Our investigation used region of interest (ROI)-to-ROI analysis to examine static resting-state functional connectivity. We identified notable differences in resting-state static functional connectivity patterns between migraineurs with and without photophobia. Red lines indicate significantly higher connectivity in patients with photophobia than in those without. Blue lines indicate significantly lower connectivity in patients with photophobia than in those without. Abbreviation; aPaHC l: Left Parahippocampal Gyrus, anterior division; Accumbens l: Left Accumbens; Cereb6 l: Left Cerebellar hemisphere lobule VI; FP l: Left Frontal Pole; HG l: Left Heschl’s Gyrus; pITG l: Left Inferior Temporal Gyrus, posterior division; pMTG l: Left Middle Temporal Gyrus, posterior division; toMTG l: Left Middle Temporal Gyrus, temporooccipital part; pPaHC l: Left Parahippocampal Gyrus, posterior division; SPL l: Left Superior Parietal Lobule; Cereb6 r: Right Cerebellar hemisphere lobule VI; pITG r: Right Inferior Temporal Gyrus, posterior division; pMTG r: Right Middle Temporal Gyrus, posterior division; and OFusG r: Right Occipital Fusiform Gyrus.

Static resting-state functional connectivity analysis showed that patients with photophobia had ten significantly different connectivities compared to patients without photophobia (Table 1, Figure 1). Of the 10 functional connections with significant differences, four were between the cerebellum and other regions, while three were between the cerebellum and the temporal lobe. These functional connections between the cerebellum and temporal lobe were significantly reduced in the group with photosensitivity compared to the group without photosensitivity.

### 3.3. Differences in Dynamic Resting-State Functional Connectivity

**Figure 2:**
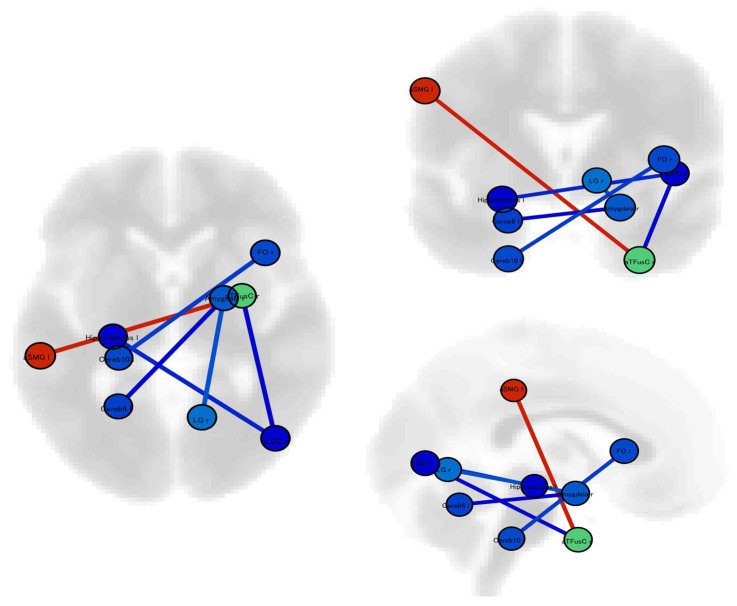
We conducted an investigation using ROI-to-ROI analysis to examine dynamic resting-state functional connectivity. Our results revealed significant differences in both static and dynamic resting-state functional connectivity between migraineurs experiencing photophobia and those without this symptom. Red lines indicate significantly higher connectivity and blue lines indicate significantly lower connectivity in patients with photophobia than in those without. Abbreviation; Cereb6l: Left Cerebellar hemisphere lobule VI; Cereb10: Left Cerebellar hemisphere lobule X; Hippocampus l: Left Hippocampus; aSMG l: Left Supramarginal Gyrus, anterior division; Amygdala r: Right Amygdala; FOrb r: Right Frontal Orbital Cortex; iLOC r: Right Lateral Occipital Cortex, inferior division; LG r: Right Lingual Gyrus; and aTFusC r: Right Temporal Fusiform Cortex, anterior division.

Dynamic resting-state functional connectivity analysis showed that patients with photophobia had six significantly different connectivities compared with patients without photophobia (Table 2, Figure 2). The functional connection between the left cerebellar hemisphere and right amygdala showed significantly lower connectivity.

## 4. Discussion

This study focused on specific resting-state functional connectivity differences in patients with migraine experiencing photophobia alone, without the confounding effects of phonophobia or osmophobia. Overall, we identified ten significant connectivity pairs in static resting-state functional connectivity and six in dynamic resting-state functional connectivity that discriminated migraineurs with photophobia from those without photophobia. Significantly reduced connectivity between the cerebellar hemisphere and the temporal region was observed in migraineurs with photophobia compared with migraineurs without photophobia in both static and dynamic analyses. These results highlight a unique pattern of connectivity associated with photophobia in patients with migraine, confirming and extending the findings of past research: Static resting-state functional connectivity analysis revealed 18 significant connectivity pairs in patients with photophobia, mainly involving the cerebellar hemispheres and regions such as the temporal occipital fusiform cortex. Dynamic resting-state analysis further revealed 16 significant connectivity pairs, primarily between the cerebellar hemisphere and other brain regions [5].

This study showed significantly different connectivity between migraineurs with and without photophobia in the cerebellum, particularly in hemispheric cerebellar lobule VI. Several studies have previously examined the functional connectivity of the cerebellum in migraineurs [7,8,9,10,11,12,13,14,15]. However, few studies have investigated lobule IV, which is thought to be involved in voluntary movement control and cognitive functions [16,17]. Individuals with migraine show increased connectivity between the hypothalamus and structures associated with the parasympathetic nervous system, such as the temporal pole, superior temporal gyrus, and cerebellar lobules V and VI [10]. The cerebellar lobules extending from VI to V process individual intensity and discomfort ratings in the facial region, whereas in studies on healthy subjects, nociceptive stimulation of the trigeminal nerve was found to activate specific cerebellar regions, including cerebellar lobule VI [9]. These studies suggest that reduced cerebellar connectivity with higher cortical areas, including the temporal regions known to be hubs of pain processing, may be related to photophobia.

Migraineurs with photophobia exhibited reduced functional connectivity between the cerebellar hemispheres and temporal lobes, including the middle temporal gyrus for static and temporal fusiform cortex for dynamic connectivity. The middle temporal gyrus can be divided into four regions: anterior (aMTG), middle (mMTG), posterior (pMTG), and sulcus (sMTG). The aMTG is predominantly associated with the default mode network, sound perception, and semantic retrieval; the mMTG is mainly associated with semantic memory and semantic control networks; the pMTG is part of the traditional sensory linguistic areas; and the sMTG is associated with gaze direction decoding and intelligible speech [18]. The temporal fusiform cortex is considered a key structure for functionally specialized computations of high-level vision such as face perception, object recognition, and reading [19]. Reduced functional connectivity of the temporal fusiform cortex may cause photophobia due to functionally specialized computations of high-level vision. Regarding the altered functional connectivity of middle temporal gyrus, it is not clear which functions are directly related to photosensitivity and may be an indirect effect.

This study has several limitations. First, the sample size was small, with only 15 patients in each group. However, as migraine patients with photophobia and without phonophobia and osmophobia are uncommon, it is difficult to recruit a large number of patients from a single center. These limitations unfortunately diminish the statistical robustness of the study, frequently necessitating suboptimal methods to establish significance and constraining the broader applicability of the findings [4]. This sample size limitation has also been observed in other functional MRI studies [10,12,14]. Further multicenter studies are required to address this issue. Additionally, no method has yet been established to determine the appropriate sample size required for fMRI studies; however, this may be resolved in the future when it becomes possible to model different sample size scenarios using computer simulations and assess the statistical power in each scenarios. Second, this study was conducted during the interictal period. Photophobia occurs primarily during the ictal phase. Brain functional connectivity during the attack period varies significantly between the preictal, ictal, and postictal phases [20,21]. It is difficult to predict precisely when a migraine attack will occur. Therefore, previous studies captured images for 30 consecutive days to obtain data during the attack period. However, scanning a large number of patients for 30 consecutive days is challenging, and finding a solution is not expected to be easy.

## 5. Conclusions

Our results suggest that an alteration in resting-state functional connectivity between the cerebellum and temporal region is specific to migraineurs with photophobia.

## Figures and Tables

**Table 1:** Significant changes in static resting-state functional connectivities in patients with vs without photophobia.

Analysis Unit	T Score	*p* Value
Left Middle Temporal Gyrus, temporooccipital part– Right Inferior Temporal Gyrus, posterior division	5.40	0.0013
Left Frontal Pole–Left Parahippocampal Gyrus, anterior division	4.15	0.0389
Left Cerebellar hemisphere lobule VI– Left Accumbens	4.10	0.0410
Left Parahippocampal Gyrus, posterior division– Right Occipital Fusiform Gyrus	3.94	0.0425
Left Middle Temporal Gyrus, temporooccipital part– Left Inferior Temporal Gyrus, posterior division	3.81	0.0321
Left Parahippocampal Gyrus, posterior division– Left Heschl’s Gyrus	−3.66	0.0474
Left Cerebellar hemisphere lobule VI– Left Middle Temporal Gyrus, posterior division	−3.71	0.0410
Left Cerebellar hemisphere lobule VI– Right Middle Temporal Gyrus, posterior division	−3.72	0.0410
Left Parahippocampal Gyrus, posterior division– Left Superior Parietal Lobule	−3.85	0.0425
Right Cerebellar hemisphere lobule VI– Left Middle Temporal Gyrus, temporooccipital part	−3.91	0.0321

**Table 2:** Significant changes in dynamic resting-state functional connectivities in patients with vs without photophobia.

Analysis Unit	T Score	*p* Value
Left Supramarginal Gyrus, anterior division– Right Temporal Fusiform Cortex, anterior division	4.69	0.0087
Right Amygdala−Right Lingual Gyrus	−3.82	0.0469
Left Cerebellar hemisphere lobule X− Right Frontal Orbital Cortex	−4.16	0.0374
Right Lateral Occipital Cortex, inferior division– Left Hippocampus	−4.53	0.0137
Right Temporal Fusiform Cortex, anterior division– Right Lateral Occipital Cortex, inferior division	−5.05	0.0034
Left Cerebellar hemisphere lobule VI−Right Amygdala	−5.20	0.0022

## Data Availability

The data supporting the findings of this study are available upon reasonable request. Please contact the corresponding author at neurologyimai@gmail.com for any additional information or specific protocols.
